# EEG Markers of Suicidal Ideation in Depressive Female Adolescents

**DOI:** 10.1192/j.eurpsy.2022.481

**Published:** 2022-09-01

**Authors:** A. Iznak, E. Iznak, T. Medvedeva, E. Damyanovich, I. Oleichik

**Affiliations:** 1 Mental Health Research Centre, Laboratory Of Neurophysiology, Moscow, Russian Federation; 2 Mental Health Research Centre, Laboratory Of Neurophysiology, Москва, Russian Federation; 3 Mental Health Research Centre, Department Of Clinical Psychology, Moscow, Russian Federation; 4 Mental Health Research Centre, Department Of Endogenous Mental Disorders And Affective Conditions, Moscow, Russian Federation

**Keywords:** female adolescents, suicidal ideation scores, quantitative EEG

## Abstract

**Introduction:**

Timely detection of suicidal thoughts is one of the ways to prevent suicide. Use the psychiatric interview only for this purpose in adolescents is often insufficient due to poor compliance. Thus, the search for objective neurophysiological markers of suicidal ideation in adolescents seems to be actual.

**Objectives:**

The aim of the study was to reveal the relationships between pre-treatment EEG parameters and intensity of suicidal ideation in depressive female adolescents.

**Methods:**

72 female depressive patients (all right-handed, age 16–25, mean 18,2±2.6 years old) were enrolled in the study. Pre-treatment total HDRS-17 scores varied from 13 to 43 (mean 26,9±7.5). Multichannel eyes closed EEG was recorded, and absolute spectral power was calculated in 8 narrow frequency sub-bands. All patients answered the inventory on intensity of suicidal thoughts. Spearman’s correlations between pre-treatment EEG parameters and suicidal ideation scores were analyzed.

**Results:**

Scores of intensity of suicidal ideation correlated positively (p<0.05÷0.01) with values of EEG alpha2 (9-11 Hz) spectral power in F7, F8, F4, C3, C4, T4, P4 and O2 EEG leads, as well as with values of EEG delta (2-4 Hz) spectral power in F7, F3 and C3 EEG leads (p<0.05).

**Conclusions:**

The intensity of suicidal ideation in depressive female adolescents associates with wide propagation of EEG alpha2, especially over the right hemisphere, and with EEG signs of decreased functional state of anterior regions of the left hemisphere. The study supported by RBRF grant No.20-013-00129a.

**Disclosure:**

No significant relationships.

